# Comprehensive Analysis of Crystalline Hydrophobic Alkylated Poly(ethyleneimine)s

**DOI:** 10.1002/chem.202500764

**Published:** 2025-05-19

**Authors:** Anand Sharadha‐Ravi Ayyar, Rodrigo J. S. Lima, Adedeji Adebukola Adelodun, Berit Lane Nagorsen, Jacob J. K. Kirkensgaard, Svemir Rudić, Heloisa N. Bordallo, Ji‐Woong Lee

**Affiliations:** ^1^ Department of Chemistry, Nano‐Science Center University of Copenhagen Universitetsparken 5, Copenhagen Ø Copenhagen 2100 Denmark; ^2^ The Novo Nordisk Foundation CO_2_ Research Center Aarhus University Gustav Wieds Vej 10C Aarhus C 8000 Denmark; ^3^ The Niels Bohr Institute University of Copenhagen Copenhagen DK‐2100 Denmark; ^4^ European Spallation Source ESS ERIC Lund SE‐221 00 Sweden; ^5^ Department of Food Science University of Copenhagen Rolighedsvej 26, Frederiksberg C Frederiksberg 1958 Denmark; ^6^ ISIS Facility Rutherford Appleton Laboratory, Chilton Didcot Oxfordshire OX11 0QX United Kingdom of Great Britain and Northern Ireland

**Keywords:** Carbon dioxide, inelastic neutron spectroscopy, ion–ion interaction, polyethyleneimines (PEIs), powder X‐ray diffraction, small‐angle X‐ray scattering, thermal analysis

## Abstract

Polyethyleneimine (PEI) is a widely used polymer in catalysis, gas separation, water treatment, drug delivery, textiles, and paper production. Carbon dioxide can act as a molecular stimulus for amine‐rich polymers such as PEIs, a property that has been exploited in CO₂‐mediated desalination. This process entails amine‐mediated CO₂ capture and the in situ formation of bicarbonate ions, which subsequently undergo ion exchange with chloride ions present in both model and real seawater. In this study, we prepared a series of alkylated PEIs with different chainlengths (C4‐C18) and degrees of alkylation (10‐40% alkylation) via simple alkylation as previously reported. The obtained hydrophobic PEI, specifically PEI‐C16, was further investigated in terms of its crystalline structure using powder X‐ray diffraction, small‐angle X‐ray scattering, thermogravimetric analysis, and inelastic neutron scattering. Based on our analysis, we conclude that the subtle structural changes of hydrophobic poly(ethyleneimine)s were induced by acid‐mediated protonation and ion exchange, while the polymer maintained its integrity across multiple desalination cycles. These findings advocate for the employment of hydrophobic poly(ethyleneimine) in CO₂‐responsive processes and carbon capture applications.

## Graphical abstract

Alkylation of polyethyleneimine (PEI) induces crystalline hydrophobic domains, critical for CO₂‐mediated desalination. Structural evolution during acid/base treatment and ion exchange is revealed by PXRD, SAXS, and inelastic neutron scattering. Findings highlight reversible domain disruption and nanostructure formation, enabling recyclable polymer performance in water purification and CO₂ capture.

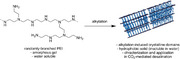

## 1 Introduction

As organic polymeric bases, polyamines are ubiquitous for many applications, ranging from biological drug deliveries^[^ ^1^, ^2^ ^]^ to carbon capture.^[^ ^3^ ^]^ Polyethyleneimine (PEI, i.e., a polymer of aziridine) has been used in various applications owing to its high loading of nitrogen bases (theoretically up to 23 mmol/g),^[^ ^4^ ^]^ hydrophilicity, and water solubility. In general, randomly branched PEls are highly accessible. In contrast, linear and dendrimeric PEIs require sophisticated preparation and synthetic strategies despite having unique properties due to their uniform chemical structures on nitrogen atoms.^[^ ^5^ ^]^

Stimuli‐responsive polymers, which alter their properties in response to environmental changes, offer a novel and potentially energy‐saving approach for water purification and regeneration.^[^ ^6^ ^]^ Various smart polymer systems can remove pollutants effectively from water using external stimuli, such as pH, CO_2_, temperature, and light, or a combination of stimuli.

Recently, we demonstrated a novel desalination process that combines CO_2_ capture and water purification using CO_2_‐responsive recyclable organic materials (also see Scheme S1).^[^ ^7^ ^]^ Leveraging the high amine density, hydrophobic alkylated poly(ethyleneimine) polymers (PEI) mediated CO_2_ uptake and ion exchange sequence. The modified randomly‐branched PEI^[^ ^8^, ^9^, ^10^, ^11^, ^12^ ^]^ successfully sequestrated chloride ion from the model and genuine seawater samples (aq NaCl of 3–35 g/L). A brine solution treated with alkylated PEIs generated soda (NaHCO_3_) and quaternary ammonium chloride on PEIs. Chloride removal was accomplished through decantation of the polymeric sediment. We found that PEI activation (acid and base treatment, Scheme S1B) was crucial to optimizing the desalination in terms of chloride removal, indicating the physical structure and molecular arrangement of alkylated PEIs may play a significant role in our CO_2_ responsive process.

Considering that the rate‐limiting process of our desalination is a CO_2_ absorption step, the PEI's performance in our desalination should be investigated beyond chloride removal efficiency and recyclability, as provided in our previous work.^[^ ^7^ ^]^ We postulated that the chemical structures, morphologies, and integrity of alkylated PEI (before and after acid treatment, CO_2_ exposure, and subsequent chloride extraction) would be crucial to improving the performance of hydrophobic PEIs. According to the literature, properties such as buffering capacity, *p*K_a_, kinetics, and reversibility of CO_2_ binding, and structural integrity of the materials are essential for the performance of CO_2_‐responsive polymers.^[^ ^13^, ^14^, ^15^ ^]^ These parameters may provide materials with adequate rate and capacity for CO_2_ uptake, thus allowing us to achieve higher performance in desalination and water purification, requiring highly energy‐efficient processes compared to the modern reverse osmosis membrane processes.^[^ ^16^, ^17^ ^]^

In this study, we employed various techniques, viz. nuclear magnetic resonance (NMR) spectroscopy, powder X‐ray diffraction (PXRD), small‐angle X‐ray scattering (SAXS), and inelastic neutron scattering (INS), to investigate the chemical and physical nature of CO_2_‐responsive hydrophobically modified PEIs. To compare their structural behavior under CO_2_ conditions with carbonic acid, we subjected alkylated PEIs to aq. HCl solution, mimicking its influence in CO_2_ capture and anion exchange sequences. Our analyses showed the impact of CO_2_ inducing spontaneous protonation and subtle disruptions in the hydrophobic domains of alkylated PEI, thus forming new nanostructures.

We hypothesize that the interactions from these hydrophobic domains are crucial to preserving the structural integrity of the polymers from CO_2_ treatments. A deeper understanding of PEI's dynamic behavior holds promise for novel applications in CO_2_ capture and simultaneous water purification and desalination using amphiphilic amine‐rich polymers.

## 2 Experimental Section

### 2.1 Materials and Synthesis

Commercially available branched polyethyleneimine (PEI): *M_w_* = 25,000 g·mol^−1^ (100% PEI, by weight) and *M*_*w*_ = 750,000 g·mol^−1^ (PEI solution, 50% (w/v) in H_2_O) were purchased from Sigma‐Aldrich. Sigma‐Aldrich also supplied Amberlite 15 hydrogen form (sulfonated polystyrene resin), bromoalkanes, and acetone (ACS reagent, ≥ 99.5%). We procured hydrochloric acid (HCl, ACS reagent, 37%) from Fischer Scientific. Sodium hydroxide (NaOH), potassium chromate (indicator), and absolute ethanol (HPLC grade) were purchased from VWR Chemicals, whereas T.H. Greyer supplied sodium chloride and potassium hydroxide. Silver nitrate was purchased from Alfa Aesar. CO_2_ (99.97% purity) was bubbled into the solution without further treatment. All the alkylation, desalination, and regeneration experiments were performed as described.^[^ ^7^ ^]^

### 2.2 Methods

**Nuclear magnetic resonance (NMR) spectroscopy**: ^1^H and ^13^C NMR spectra were recorded with Ultra Shield Plus 500 spectrometers at 500 MHz (^1^H spectra) and 126 MHz (^13^C spectra) on a Bruker Avance 3 spectrometer with a BBFO probe. The obtained spectra were evaluated using MestReNova (14.2.0) software. The analysis solvents: deuterated chloroform (CDCl_3_: 7.26 ppm (^1^H NMR) and 77.0 ppm (^13^C NMR)) and deuterated water (D_2_O: 4.80 ppm (^1^H NMR)) were purchased from Sigma‐Aldrich. All chemical shifts (δ) were measured in parts per million (ppm) relative to tetramethyl silane (TMS).

**Low‐field benchtop (Tveskaeg Bench‐top) nuclear magnetic resonance spectroscopy**: To determine the concentration of salts pre‐ and post‐desalination experiments, permanent low magnetic field (in the range 1–2 T) as opposed to the conventional high field ^1^H and ^13^C NMR (range 5–25 T) magnet was used as a benchtop NMR. It uses a multinuclear sensor to analyze various nuclei (^23^Na, ^35^Cl, and ^39^K) and provide their ionic concentrations within a given solution under investigation.^[^ ^18^ ^]^

**Differential scanning calorimetry (DSC)** was used to measure the melting temperature of **PEI‐C16‐33%** and ‐**20%**. PEI residues were heated using a TA Instrument DSC250 differential scanning calorimeter at 10 °C·min^−1^ from 25 °C to 200 °C under a nitrogen atmosphere with a flow rate of 50 mL·min^−1^.

**Thermogravimetric analysis (TGA)** coupled with infrared spectroscopy (FTIR): A Netzsch TG 209 F1 Libra (Netzsch, Germany) was used for TGA, in conjunction with a Bruker Fourier Transform Infrared (FTIR) spectrometer. PEI‐C16 samples were placed in an Al₂O₃ crucible and heated from 28 °C to 800 °C at a rate of 10 °C/min under a nitrogen flow of 20 mL/min. The FTIR spectrometer recorded gas‐phase spectra every 3 °C within a range of 650–4400 cm^−1^.

**Small‐angle X‐ray scattering (SAXS) and wide‐angle X‐ray scattering (WAXS) experiments** for PEI residues were performed using the GANESHA‐SAXS/WAXS instrument from SAXS‐LAB (Denmark), equipped with a 100XL+ microfocus sealed X‐ray tube CuKα radiation (*λ* = 1.54 Å) (Rigaku, Japan) with Kirkpatrick–Baez focusing mirror system and a 2D 300k Pilatus detector from Dectris (Switzerland). The measurements were carried out at two distances (50 mm and 350 mm) by varying the distance between the sample holder and the detector.

**Powder X‐ray diffraction** (PXRD) of PEI residues was analyzed using a Bragg–Brentano diffractometer setup having a CuKα (1.54 Å) source on a BRUKER D8 Discover and a LYNXEYE detector. All data (except for **PEI** = 2°− 60°) were collected in the range of 3°−60° 2θ. The scattering vector q and d‐spacing values were calculated using the following equations:

(1) $$d=\frac{\lambda }{2\sin\;(\theta )}$$

(2) $$q=\frac{2\Pi \;}{d}\;$$

where *λ* = 1.54 Å is the X‐ray wavelength, and *θ* is half of the scattering angle.

**Inelastic neutron spectroscopy (INS)** was used to investigate hydrogen atoms in local comb‐like polymeric structures 1.011 g of **PEI‐C16‐33%**, between 20 and 4000 cm^−1^. Spectra were recorded at 20 K to minimize the thermal motion of the adsorbed molecules and structure, using the inverse geometry time‐of‐flight spectrometer (TOSCA) at the ISIS Facility in the United Kingdom.^[^ ^19^ ^]^ Data were reduced using the MANTID software package.^[^ ^20^ ^]^ As part of the data reduction, the spectra are automatically normalized in relation to the proton beam current and the signal coming from an empty aluminium subtracted. As the incoherent cross section (σ_H_) is about 80 times larger than the other atoms in **PEI‐C16‐33%**, the spectra measured on TOSCA are dominated by vibrational motions of the hydrogen atoms.^[^ ^21^ ^]^

**Density functional theory (DFT)** calculations were performed using Gaussian16 at the B3LYP/6–311G level of theory, in order to optimize the atomic positions of the comb‐like polymeric structure idealized for **PEI‐C16‐33%**.^[^ ^21^, ^22^ ^]^ Such optimized structure was then used in order to calculate harmonic vibrational frequencies associated with it. In order to compare derived theoretical spectra with the experimental INS measurements we needed to recalculate the intensity of its vibrational modes, using derived atomic displacements, which was done with the help of AbINS plugin within MANTID software^[^ ^20^, ^23^ ^]^ The Gaussian output file was directly used as input, and the spectral broadening corresponds exclusively to the instrument resolution implemented in AbINS.^[^ ^20^, ^23^ ^]^ The combination of INS experimental spectra with DFT calculations allows assigning which molecular groups contribute most to each part of the INS spectra, elucidating the mechanism of hydrogen bonding interactions.^[^ ^24^ ^]^

## 3 Results and Discussion

**Figure 1:**
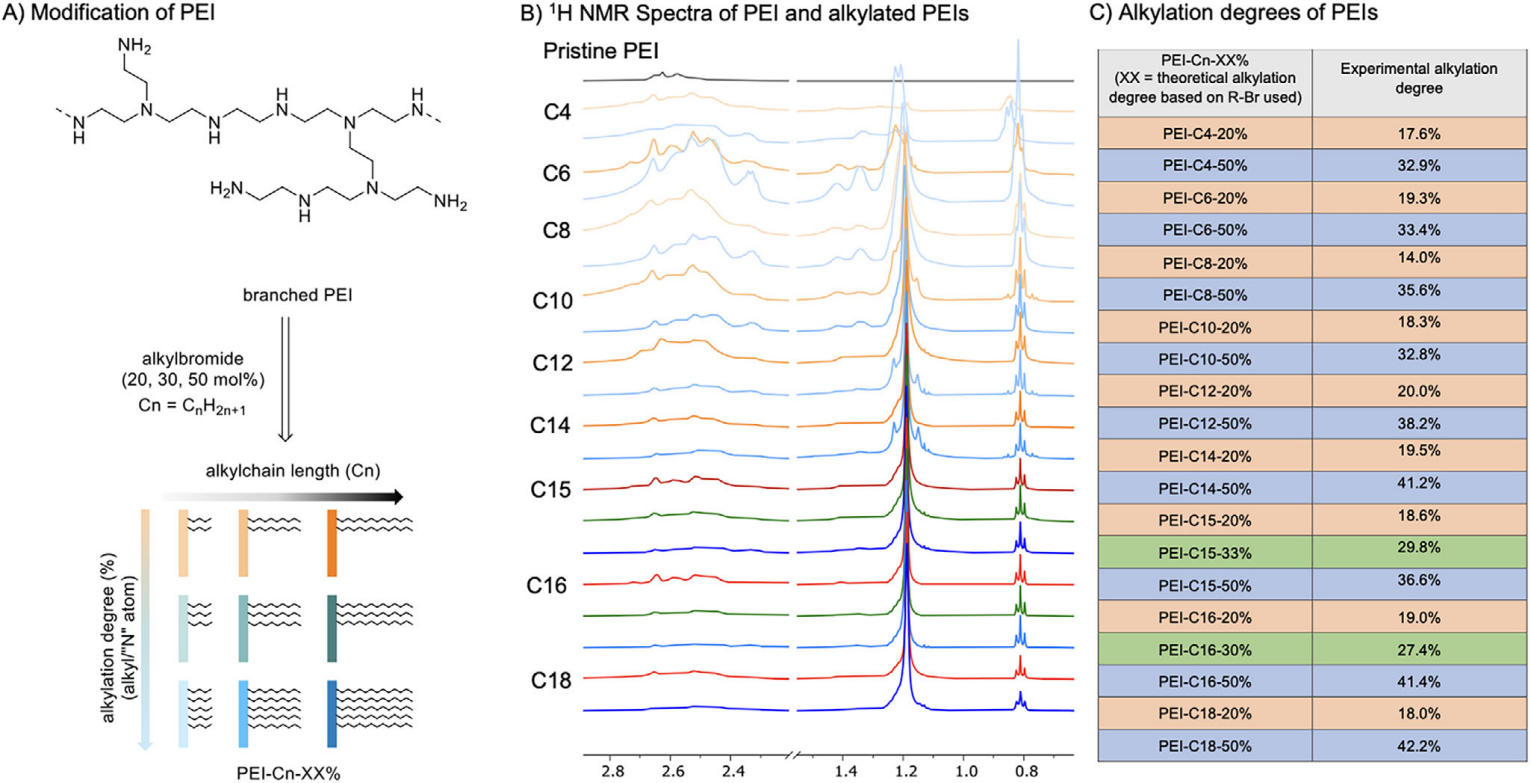
A) A representative chemical structure of the studied poly(ethyleneimine) (PEI) polymer (750 K) and its modification via alkylation. B) Selected ^1^H NMR spectra of the obtained alkylated PEIs with different alkyl chain lengths and alkylation degrees (PEI‐Cn‐XX%). C) Summary of alkylation degree of experiments (expected alkylation degree based on the stoichiometry of used alkyl halides, and experimental alkylation degree determined by ^1^H NMR spectroscopic analysis).

Branched PEI is a random polymer that is available as an aqueous solution. Using aqueous PEI solution (750,000 average molecular weight), modified PEI was prepared with different alkyl chain length and alkylation degree (Figure 1A). Owing to the hydrophobicity of the obtained polymers, alkylation degree of PEI samples were precisely determined by ^1^H NMR analysis (Figure 1B), showing slight difference between intended and observed alkylation degree particularly for higher alkylation degree (Figure 1C), which can be ascribed to the steric congestion occurring at high alkylation degree and longer alkyl chain substitution. Nonetheless, we selected C16 alkylation as a standard method in this study owing to its high reproducibility and optimal performance in our CO_2_‐mediated desalination. Alkylation of PEI with C16‐Br yielded powdery PEI‐C16, thus enabling its facile characterization and analysis. Moreover, it is insoluble in polar solvents (water and ethanol) in a wide (2–14) pH range (Scheme S1D). This change in solubility and viscosity after the alkylation reactions of PEIs indicated the presence of localized structural order within modified PEI with more than 20% alkylation degree, which is determined by ^1^H NMR spectroscopy. The DSC analysis showed no glass transition temperature for these polymers within 25–200 °C (Figure S1). Melting occurred at 123 and 133 °C for PEI‐C16‐33% and PEI‐C16‐20%, respectively, ascribable to the additional hexadecyl side chains on the PEI backbone (See Supporting Information TGA and DSC analysis).

### 3.1 Crystalline Phase Transformation of Amorphous PEI

**Figure 2:**
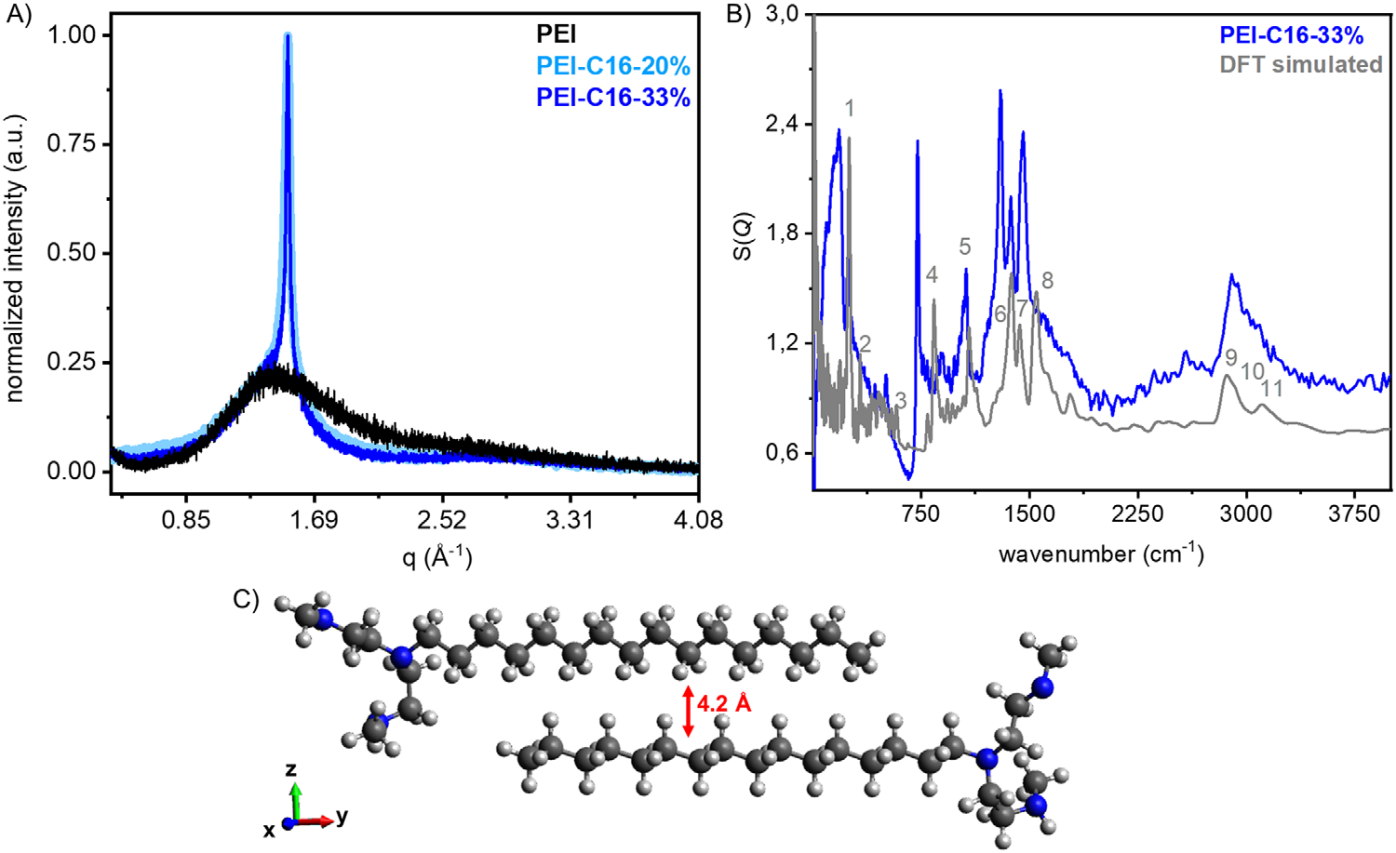
A) Powder X‐ray diffraction patterns (normalized) of **PEI‐C16‐33%** and **PEI‐C16‐20%**; q (1.5 Å) and d‐spacing (4.2 Å) values were calculated using Equations (1) and (2); B) Experimental and theoretical inelastic neutron scattering spectra of **PEI‐C16‐33%** at 19 K; C) Backbone of truncated comb‐like polymer structures (**PEI‐C16‐33%**) used in the DFT calculations.

To investigate the crystallinity of PEI and PEI‐C16 powder, we employed PXRD analysis to corroborate the presence of regions of coherent diffraction within the samples. PEI (after drying the commercial aqueous solution of PEI, that is, PEI in Figure 2A, black) shows an amorphous state with a broad diffraction peak around 2θ ≤ 20°, resembling the liquid state with no ordered structure. Upon alkylation, the polymers (PEI‐C16‐33% and PEI‐C16‐20%) exhibited a sharp diffraction peak at 2θ ≤ 21.39° with a 4.2 Å d‐spacing (Figure 2A). It indicated the formation of coherently diffracting regions, for example, crystallites or domains of hexadecyl (C16) chains within the polymer, potentially due to the hydrophobic interaction between two C16 chains (Figure 2C).^[^ ^8^, ^25^ ^]^

Using inelastic neutron spectroscopy (INS) coupled with DFT calculations, where two units of PEI with their C16 chains—representing a condition with a high degree of ordering and rigidity—were considered to represent the PEI‐C16‐33% sample, revealed that C16 hydrophobic chain interactions may exist as comb‐like domain structures within alkylated PEI (Figures 2B,C).^[^ ^26^ ^]^ This conclusion is based on the fact that even if the simple approach used here shows that the theoretical results agree well with the experimental spectra, one also observes large discrepancies ∼ 1500 cm^−1^. Vibrations in this region are related to the sample's stiffness and agreed well with ^1^H NMR analyses of PEI‐C16‐33%.^[^ ^7^ ^]^

In more detail, the prominent bands corresponding to symmetric and asymmetric C─H stretching were distinctly observable at ∼ 2900 cm⁻¹ in the experimental spectra, accompanied by an N─H stretching mode at ∼ 3100 cm⁻¹. The strong intensity in the 1000–1700 cm^−1^ frequency range is ascribed to vibrations consisting of coupled angular deformations involving hydrogens in the C16 chains (CH_2_ twisting, CH_2_ wagging, and CH_2_─CH_3_ scissoring, Figure 2B and Table S1). Additionally, vibrations between 700 and 1000 cm^−1^ are assigned to CH_2_ – rocking of the C16 chains, while the low‐frequency modes from 150 to 700 cm^−1^ to CH_2_ – torsions of the C16 chains, CH_3_ torsions, and C─C scissoring, thus causing displacements between the carbons. Finally, the sharp peaks at 187 cm^−1^ (PEI‐C16‐33%) and 252 cm^−1^ (mode 1, DFT simulated, gray) are assigned to methylene (CH_2_) torsions. In this region, differences of about 100 cm^−1^ were observed between the experimental and theoretical peaks. We postulate that this is a consequence of the simplified structure used for PEI derivatives. Table S1 summarizes the calculated and experimental vibrations.

Based on the scattering results, where the coherent length of molecularly ordered regions and a possible comb‐like orientation of C16 chains may result in high integrity of alkylated PEIs, thus enabling their applications in CO_2_‐mediated desalination. Next, we decided to follow up on the crystalline domain step‐by‐step by mimicking the desalination procedure (Scheme S1C) and analyzing their PXRD patterns.

### 3.2 Disrupting Coherent Length and Emerging New Nanostructures

**Figure 3:**
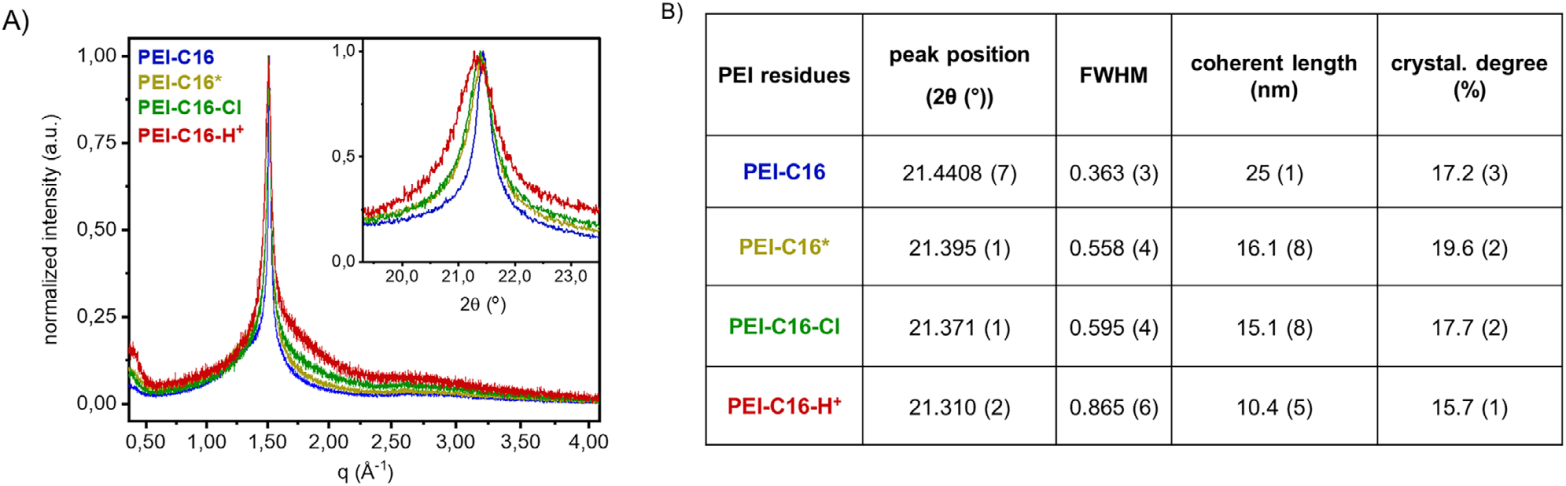
A) Powder X‐ray diffraction patterns (normalized) of PEI‐C16, protonated form (PEI‐C16‐H^+^), activated form (PEI‐C16*), and after desalination in aq NaCl solution (PEI‐C16‐Cl). The inset shows the broadening effect of FWHM; B) Table: The peak position, full width at half maximum (FWHM), and crystallinity degree based on the PXRD patterns of **PEI‐C16‐33%** samples. These evaluations involved removing the amorphous contribution and using the Pearson VII function adjusted in the Origin Lab software package. In addition, coherent length estimation was facilitated by applying the Scherrer equation.

The interplay of polymer chain interactions can create rigid (coherent) and soft (amorphous) regions that determine a polymer's solid, liquid, or molten state. The degree of crystallinity and amorphous content within PEI‐C16 was estimated using the method applied by Dome et al.^[^ ^27^ ^]^ The amorphous and crystalline components were calculated as a ratio between the area corresponding to the coherent length and the total area of the PXRD pattern (Figure 3A).^[^ ^27^ ^]^ Our analysis shows that PEI‐C16, as a switchable polymer, formed nanostructures when subjected to variable pH and temperature conditions, presumably due to the prolonged acidic treatments with an acid, for example, CO_2_.^[^ ^28^, ^29^ ^]^

Because our CO_2_‐responsive polymer exhibited pH‐sensitive behavior,^[^ ^7^ ^]^ we treated the polymer with HCl (Scheme 1B, PEI‐C16‐H^+^). After the acid treatment, the residue was neutralized with a base (NaOH or KOH), which allowed the activated sample PEI‐C16*. The desalination experiments were conducted with aqueous NaCl solution (NaCl = 3 g/L). Subsequent analyses compared PEI‐C16‐H^+^ with residues derived from desalination experiments in NaCl solution (PEI‐C16‐Cl) to elucidate the chemical and physical properties of the polymers at each desalination stage.

When subjected to pH variations, the PXRD analysis of the polymers revealed disruption in the coherent length of alkylated PEI. All PEI‐C16 samples (Figure 3A): pristine (PEI‐C16), activated (PEI‐C16*), post‐desalination (PEI‐C16‐Cl), and acid‐treated (PEI‐C16‐H^+^) samples exhibited an X‐ray diffraction peak at 2θ ≈ 21.4°, corresponding to a 4.2 Å distance commonly attributed to the crystallization of comb‐like polymers with alkyl side chains.^[^ ^8^, ^26^ ^]^ According to Shi et al.,^[^ ^8^ ^]^ the crystallization of alkyl side chains occurs in a hexagonal lattice with an approximate distance of ∼ 4.2 Å between the alkyl chains. We calculated the variations in full width at half maximum (FWHM) values of crystallite size, which implied changes in the degree of finite coherent length based on the acidic treatment and desalination experiments (Figure 3B). The normalized PXRD patterns provide a clearer visualization of the broadening effect of FWHM values. To better represent this effect, the table (Figure 3B) lists the obtained FWHM values for the diffraction peaks at 2θ ≈ 21.4°. Utilizing the Scherrer equation (Equation 3),^[^ ^30^ ^]^ the coherent length of the polymers was calculated, and the increased FWHM of the diffraction peak corresponded to the decreased coherence, indicative of a lower degree of molecular order (Figure 3B).^[^ ^31^ ^]^

(3) D=kλ/βcosθ

where *D* is the crystalline domain size, *λ* is the wavelength of the radiation (nm), *θ* is the angle of the considered Bragg reflection, *β* is the width on a 2θ scale, and *k* is a dimensionless constant (close to unity). Hence, larger FWHM values indicate a small long‐range order. PEI‐C16‐H^+^, therefore, exhibited a lower coherent length than pristine PEI‐C16. The same trend was observed with the crystalline content.

The solid‐state packing of comb‐like polymers is intricately associated with the segregation process by crystallizing C16 side chains and amorphous chains. Consequently, driven by the covalent bonds between the side and main chains, this segregation manifests an alternating arrangement of amorphous and crystalline contents.^[^ ^32^ ^]^ By examining the crystalline content and coherent length, we concluded that the acid treatment suppresses the segregation of alkyl side chains into forming long‐range order and crystalline content in the solid state. However, this effect can be reversible upon neutralization, yielding the activated sample (PEI‐C16*). The decrease in the coherent length observed in PEI‐C16‐Cl (Figure 3B) can be attributed to interactions involving the chloride moiety incorporated within the polymeric structure as ammonium chloride.

**Figure 4:**
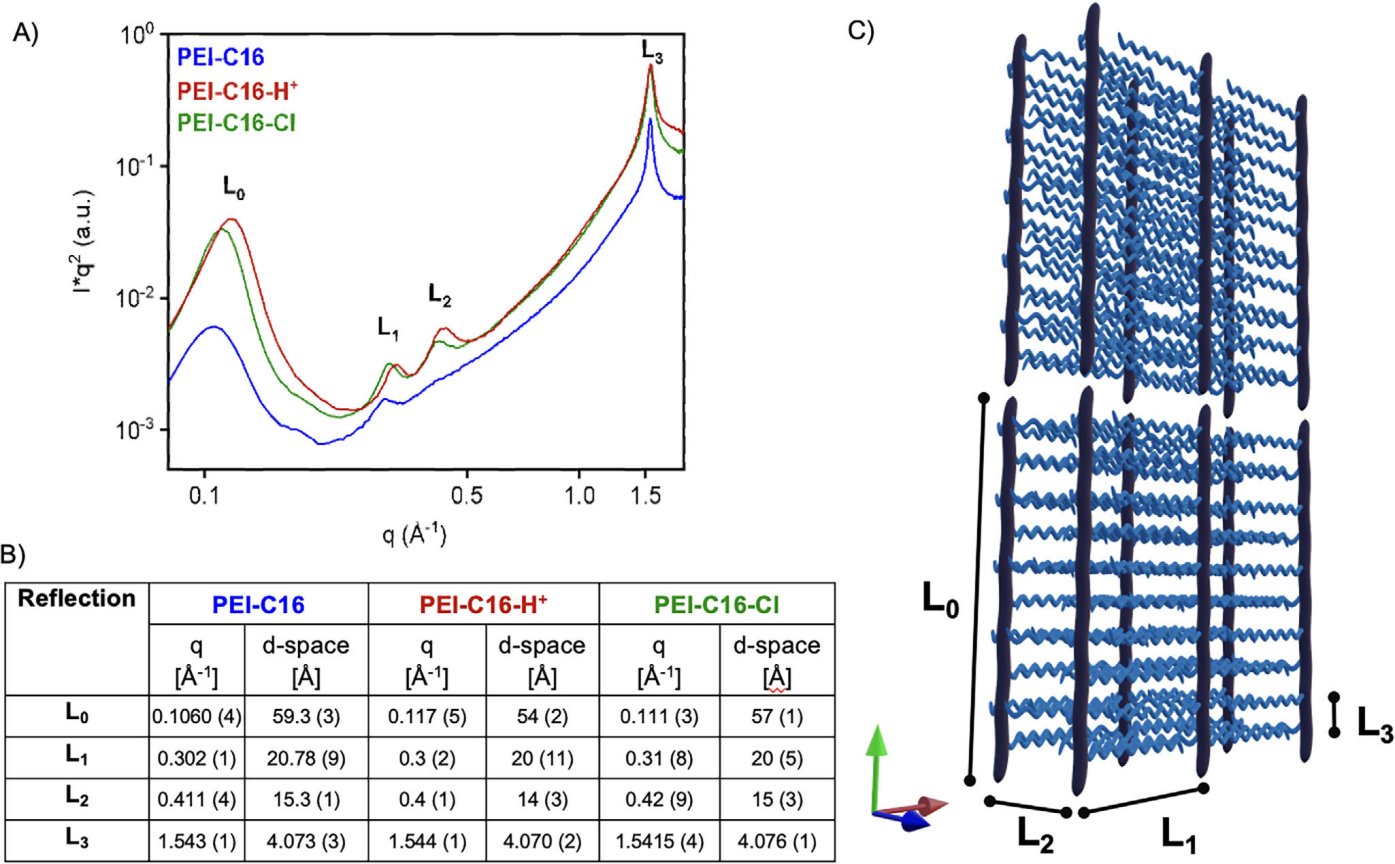
A) SAXS profiles of PEI‐C16, protonated form (PEI‐C16‐H^+^), and after desalination in aq NaCl solution (PEI‐C16‐Cl); B) Table: Bragg reflections for the PEI‐C16 samples observed in the SAXS results. Peak positions were obtained by Gaussian fits using custom MATLAB code and the Curve Fitter tool, with background estimation based on the method described by Brückner.^[^ ^33^ ^]^ The table also includes the uncertainties associated with the peak positions; C) A 3D‐comb‐like schematic model for the PEI‐C16 structure, highlighting the interlayer as alkyl chain distances. The illustration is not drawn to scale and should be considered solely for the purpose of qualitative comparison, where the red column (longitudinal section, L_0_) and gray chains (L_1_) represent the PEI backbone acting as a support and the C16 side chains, respectively.

To determine the structural change due to CO_2_‐induced protonation, we examined PEI samples using small‐angle X‐scattering (SAXS, Figure 4A). SAXS results indicate the structural characteristic changes of PEI‐C16 samples upon activation, acidification, CO_2_ capture, and ion exchange (desalination). The reflections observed in SAXS at high angles correspond to the ordering pattern of the C16 side chains with different treatments – acidification, basification, and the CO_2_‐mediated chloride removal process. The low‐angle scattering reflections indicate the spatial repetitions associated with the layered structure, with the main chains occupying distinct crystalline domains.^[^ ^26^ ^]^ A reflection at approximately *q* = 1.5 Å⁻¹ or d‐space = ∼ 4.2 Å appears in all samples, representing the periodicity of the C16 chain groups (Figure 4A).^[^ ^8^, ^26^, ^31^, ^32^, ^33^ ^]^ The same spatial spacing was observed in the PXRD analysis (Figure 3A), suggesting a strong self‐organization interaction between these side chain groups, possibly induced by hydrophobic effects during the synthesis and postsynthetic treatments in for activate and prufication of alkylated PEIs, suggesting the role of ion association and pH‐dependent charge distribution in modulating the structures of the polymers.^[^ ^32^ ^]^

Figure 4B summarizes the reflections observed by SAXS for various PEI‐C16 samples. Using a structural model for a similar comb‐like polymer (Figure 4C), we assigned 24 reflections to the distances of repeating interlayers. Following our PXRD analysis, protonation with a strong acid (PEI‐C16‐H^+^) contracted the interlayer distances more than in the pristine sample (PEI‐C16). This contraction is less pronounced in the post‐desalination sample (PEI‐C16‐Cl), suggesting that ion association and pH influence this behavior. The contraction reaches 12% for distance L_0_ and 8% for L_1_ in PEI‐C16‐H^+^ and 7% for L_0_ and 3% for L_1_ in the PEI‐C16‐Cl. A small reflection peak was observed for the interlayer distance L_2_ in PEI‐C16. This observation suggests that the acid treatment induces an alignment between layers, as observed in smectic‐like systems,^[^ ^26^, ^34^ ^]^ which persists even after CO_2_‐mediated desalination (Figure 4A). However, no alteration of distance is observed between the C16 side chains, reinforcing the robustness of this interaction upon protonation and the presence of ions and concomitant changes in the charge distributions on the amines of PEI backbone. These results, and the proposed model, served as the foundation for the 3D construction of the **PEI‐C16** system in DFT calculations and further encouraged investigation of the thermal stability of the **PEI‐C16** at each stage of the desalination process.

### 3.3 Influence of CO_2_‐Induced Protonation on PEI's Thermal Stability

When alkylated PEI is protonated in aq. HCl solution (0.1 N, PEI‐C16‐H^+^), its cationic charge density increases, which may induce morphological alterations within the alkylated PEI.^[^ ^28^, ^35^ ^] 1^H NMR spectroscopy verified the protonation of the polymers qualitatively (Figures S5), however, without quantification of protonation degree.

**Figure 5:**
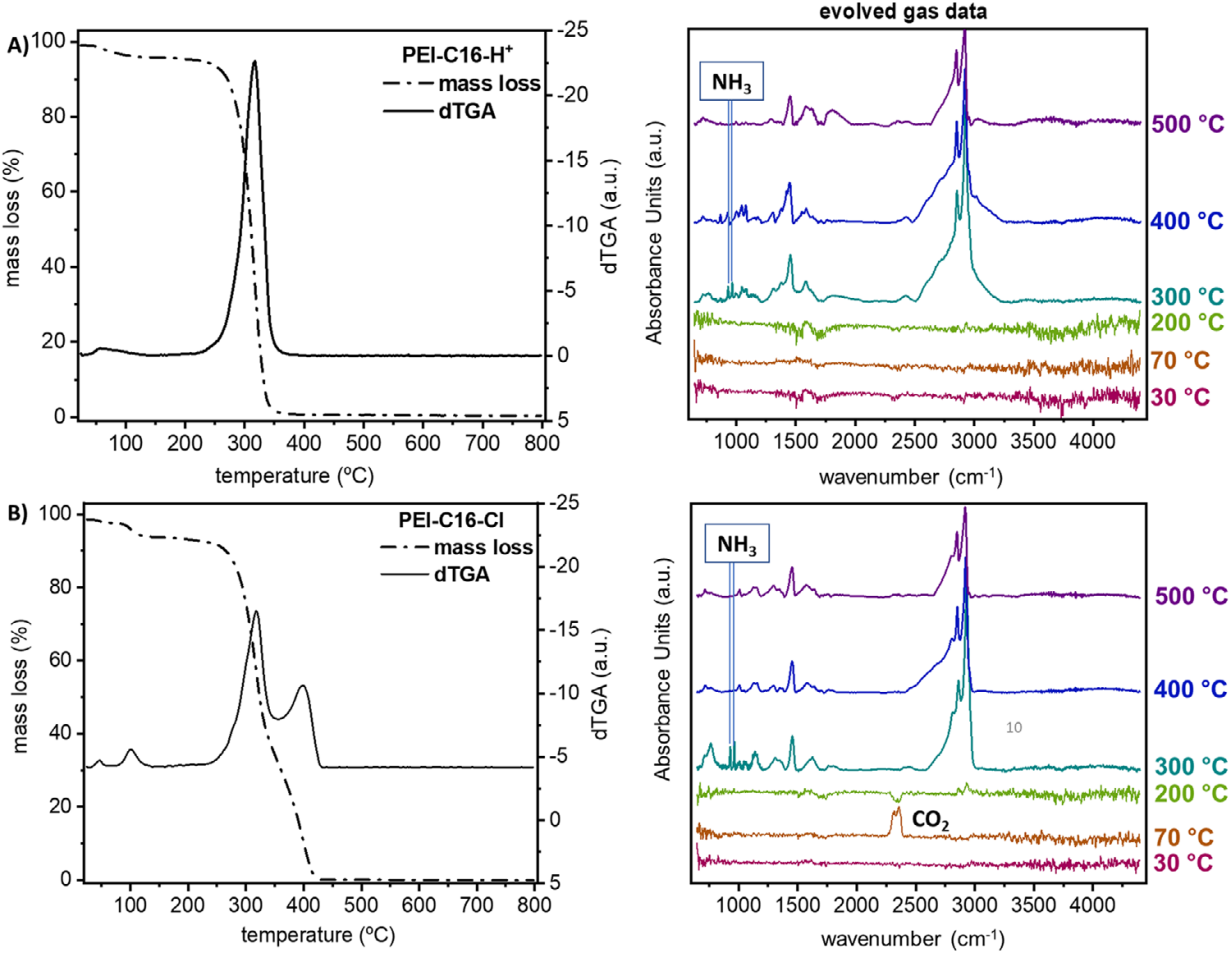
Influence of acid and CO_2_‐induced protonation on thermal properties of PEI‐C16. TGA and dTGA (derivative of TGA data) results for protonated residues from A) aq. HCl and B) desalinated residues (after desalination of aq. NaCl (3 g/L) solution; PEI‐C16‐Cl) along with their IR spectra of evolved gases at six different temperatures (30 °C, 70 °C, 200 °C, 300 °C, 400 °C, and 500 °C).

Thermogravimetric analysis coupled with infrared spectroscopy (TGA‐FTIR; under nitrogen atmosphere) was conducted on PEI samples post‐acid treatment and desalination experiments to further elucidate the protonation degree and the structural stability. We observed that PEI‐C16 and PEI‐C16* have comparable TGA profiles with complete decomposition at 420 °C (Figure S7).^[^ ^7^ ^]^ TGA and the first derivative of TGA curves (dTGA) showed > 95% mass loss occurred between 240 and 350 °C, thus suggesting that > 90% of PEI amines were protonated in the aq. HCl solution (Figure 5A). The proportional correlation of the coherent length of the C16 polymeric chain and the thermal stability can be concluded as follows: acid treatment and protonation reduce the thermal stability of PEI‐C16, following the smaller coherent length and crystalline index of PEI‐C16 as confirmed by PXRD analysis.

After CO_2_‐mediated desalination, the PEI‐C16‐Cl residue (containing both chloride and bicarbonate counter anions) was collected and subjected to TGA analysis (Figure 5B). A two‐stage mass loss was observed; the initial mass loss followed decomposition similar to that of protonated PEI (PEI‐C16‐H^+^), whereas the subsequent phase (after 350 °C) aligned with the thermal decomposition profile of PEI‐C16 and PEI‐C16* (Figure S7). This TGA result confirms that CO_2_‐mediated desalination conditions led to partial protonation of PEI‐C16, possibly forming ammonium salts (ammonium chloride and bicarbonate). Thus, investigating the exact distribution (homogeneity of anions) would be a critical future study.

Moreover, the TGA integrated with an FTIR gas analyzer identified the gases evolved in the mass loss region. The FTIR spectra of the evolved gases were assessed at six temperatures (Figures 5A and 5B, right side), selected based on the temperature range corresponding to the mass loss observed in each residue. The TGA‐FTIR spectra for PEI‐C16‐Cl displayed an absorbance peak at 2345 cm^−1^ at 70 and 200 °C, assigned to residual CO_2_, which disappeared at elevated temperatures. As the polymer started degrading at 300 °C, two distinct peaks emerged at 942 and 966 cm^−1^, ascribed to ammonia (Figures 5B and S8).^[^ ^36^, ^37^ ^]^ The ammonia‐associated absorbance peaks disappeared at 400 and 500 °C in the PEI‐C16‐Cl spectra, suggesting quaternary ammonium cation decomposition.

After confirming the thermal stability of the hydrophobic PEIs before and after protonation with strong (HCl) and weak acids (H_2_CO_3_), we studied the impact of recyclability on their desalination performance.

### 3.4 Recycling and Characterization of PEI‐C16

**Figure 6:**
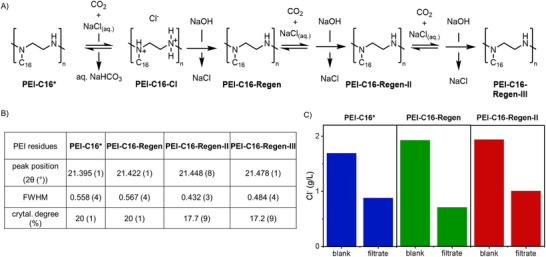
A) Scheme for multiple desalination and regeneration cycles of PEI‐C16 and desalination data in terms of chloride ion concentration for each step. B) Table: The peak position, full width at half maximum (FWHM), and crystallinity degree of the X‐ray diffraction (PXRD) patterns were assessed for desalination residues of PEI‐C16‐33% during three cycles of desalination and regeneration. These evaluations involved removing the amorphous contribution and using the Pearson VII function adjusted in the Origin Lab software package. C) Chloride reduction performance of PEI‐C16* after three regeneration cycles (fresh batch of aq NaCl (3 g/L) was used for desalination after each regeneration step).

The polymer residues after CO_2_‐mediated desalination can be regenerated using a strong base (aq NaOH solution, 0.1 N) and subjected to the next round of CO_2_‐mediated desalination with aq NaCl solution (Figure 6A). This PEI‐C16 regeneration and recycling was repeated thrice and monitored for structural variations using PXRD and peak analyses. A slight broadening of diffraction peaks was observed after the first desalination in the PXRD data of PEI‐C16‐Cl (Figure S10). However, all stages of regeneration (after base and acid treatment) revealed no significant broadening in the three regenerated residues compared to PEI‐C16*. A similar trend was observed in the residue after the first regeneration cycle, where the crystallinity degree of PEI‐C16‐Regen (19.8%) was similar to that of PEI‐C16* (19.6%). After the second and third regeneration steps, we observed a slight decrease in crystallinity to 17.7% and 17.2%, respectively (Figure 6B). Despite these subtle variations in crystalline content, alkylated PEI showed consistent chloride removal performance across multiple regeneration cycles (Figure 6C). Also, TGA and NMR spectroscopy confirmed that the structural and molecular integrity of the polymer remained intact (Figures S12 and S13), highlighting the significant role of alkylation in sample recovery without compromising the desalination performance.

## 4 Conclusion

We verified that the alkylation of amorphous PEI polymers can induce crystalline domains. Notably, we deduced that these domains could be responsible for the desired insolubility in water, which enabled easy recovery of alkylated PEI residue after desalination of the model and genuine seawater (via standard filtration). Spectroscopic analysis of post‐protonated PEI residues further revealed disruption in the coherent length and simultaneous formation of nanostructures due to protonation of the polymer under CO_2_ in aq. salt solutions. PXRD data helped understand the changes in the segregation process of crystalline C16 (alkyl) chains within PEI. Moreover, it formed the basis for DFT calculation that assisted us to construct a 3D truncated backbone using PXRD and SAXS data. The experimental INS and calculated spectra agreed, even if the approach used here is very simple, enabling a better understanding of the assignment of the main hydrogen vibrational modes of soft materials based on PEI. Finally, the observed trend in diffraction peaks of regenerated residues aligns with alkylated PEI's consistent chloride removal performance after multiple regeneration cycles. In a similar approach, these materials can be employed in water purification for anion exchange besides chloride,^[^ ^38^ ^]^ while harnessing the captured CO_2_ as a byproduct of the process, thus contributing to carbon capture and sequestration.

## Supporting Information

Supplementary text Scheme S1, and Figures S1–S14 are found in Supporting File.

## Author Contributions

The project was conceptualized by Heloisa N. Bordallo and Ji‐Woong Lee. The manuscript was written by all the authors. All the spectroscopic experiments and analysis were conducted by Anand Sharadha‐Ravi Ayyar, Rodrigo J. S. Lima, Ji‐Woong Lee, and Heloisa N. Bordallo. DFT calculation of the theoretical INS spectrum was conducted by Rodrigo J. S. Lima. Svemir Rudić performed INS experiments. Desalination experiments and thermal analysis were performed by Anand Sharadha‐Ravi Ayyar. All authors have approved the final version of the manuscript.

## Conflict of Interests

The authors declare no conflict of interest.

## Supporting information



Supporting Information

## Data Availability

The data that support the findings of this study are available in the supplementary material of this article.
