# Supplementary material for: Comprehensive Analysis of Crystalline Hydrophobic Alkylated Poly(ethyleneimine)s
Source: Chemistry. 2025 May 19;31(34):e202500764. doi: 10.1002/chem.202500764 (PMC12172576; doi:10.1002/chem.202500764)
Supplement: Supplementary file 1 — Supporting Information [file CHEM-31-e202500764-s001.pdf]

Supporting Information For

Comprehensive Analysis of Crystalline Hydrophobic Poly(ethyleneimine)s

Anand Sharadha-Ravi Ayyar,[1,3‡] Rodrigo J. S. Lima,*[1‡] Adedeji Adebukola Adelodun,[1,3] Berit Lane Nagorsen,[1] Jacob J. K. Kirkensgaard,[2,4] Svemir Rudić,5 Heloisa N. Bordallo,*[2] Ji-Woong Lee*[1,3]

[1]Department of Chemistry, Nano-Science Center, University of Copenhagen, Universitetsparken 5, Copenhagen Ø, 2100, Denmark

[2]The Niels Bohr Institute, University of Copenhagen, DK-2100 Copenhagen, Denmark; European Spallation Source ESS ERIC, SE-221 00 Lund, Sweden

[3]NovoNordisk CO_2 Research Center, Aarhus, Denmark

[4]Department of Food Science, University of Copenhagen, Rolighedsvej 26, 1958Frederiksberg C, Denmark

[5]ISIS Facility, Rutherford Appleton Laboratory, Chilton, Oxfordshire OX11 0QX, UK

*Correspondence to: bordallo@nbi.ku.dk; jiwoong.lee@chem.ku.dk

[‡]These authors contributed equally.

KEYWORDS: Crystalline domains, ion-ion interaction, powder X-ray diffraction, small-angle X-ray scattering, inelastic neutron spectroscopy, thermal analysis, DFT calculations

This PDF file includes:

Supplementary Text
Figures S1-S14

Supplementary Text

Several peaks are present in the experimental and DFT-calculated INS spectra. High intensity bands in the experimental spectra were observed at ~ 2900 cm^{-1}, ~ 1370 cm^{-1}, ~ 1065 cm^{-1}, ~ 729 cm^{-1}, ~ 515 cm^{-1} and ~ 175 cm^{-1}. The main experimentally measured bands were also present in the calculated spectrum. However, there were some clear differences between experiment and theory. For example, the position of the intense experimental peak at ~ 187 cm^{-1} is found in the theoretical spectrum at 252 cm^{-1}. It was possible to qualitatively identify some characteristics of the theoretical and experimental INS peaks. In Table 1, descriptions of the modes selected for the experimental INS spectrum and calculated by DFT are listed.

Table S1. Assignment of the main vibrations observed in the INS spectra and calculated by DFT using Gaussian software. The modes are numbered in Figure 1B. Assignment was based on the modes as well as results from the literature.[1]

Mode	Experimental (cm^{-1})	Calculated (cm^{-1})	Assignment – main contributions
1	187	252	CH_2 – Torsion – C16 chain
2	250	330	CH_3 – Torsion
3	505	583	C-C – Scissoring / C-C – Skeletal displacements
4	727	840	CH_2 – Rocking – C16 chain
5	1063	1076	CH_2 - Twisting
6	1298	1376	CH_2 - Twisting
7	1368	1433	CH_2 – Wagging – C16 chain
8	1459	1546	CH_2, CH_3 – Scissoring – C16 chain
9	2897	2853	CH_2 – CH_3 – Symetric – C16 chain
10	3005	2918	CH_2 – CH_3 – Assymetric – C16 chain
11	3189	3129	N-H - Stretching

A) PEI-Alkylation

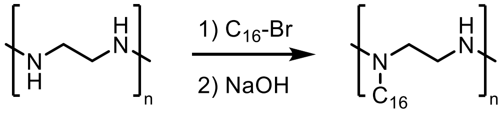

PEI **PEI-C16**

D)

solubility	PEI-25K	PEI-750K	PEI-750K-C16
water	soluble	soluble	insoluble
EtOH	soluble	soluble	insoluble
THF	soluble	insoluble	soluble
chloroform	insoluble	insoluble	soluble

(**25K** = 25000 g·mol^{-1}; **750K** = 750000 g·mol^{-1})

B) Activation using acid/base workup

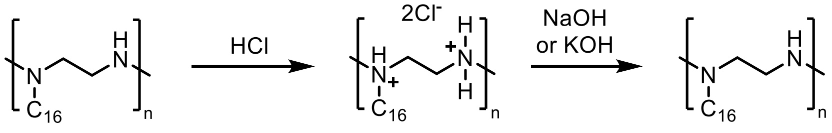

PEI-C16 **PEI-C16-H$^+$** **PEI-C16***

C) CO_2-Mediated Desalination and Regeneration

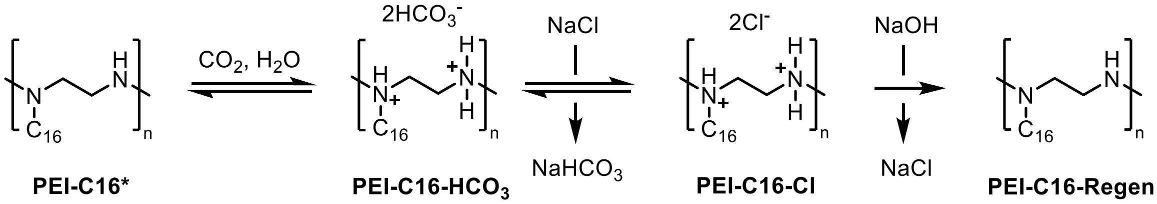

PEI-C16* **PEI-C16-HCO$_3$** **PEI-C16-Cl** **PEI-C16-Regen**

Scheme S1. Chemical structures of studied poly(ethyleneimine) (PEI) polymers (750K, alkylation degree 33% compared to the nitrogen content) (A) alkylation step, (B) activation step, and (C) CO_2-mediated anion exchange step and regeneration of the PEI; (D) Table: Solubility test was conducted in gram scale for two different molecular weight of PEI in selected solvents.

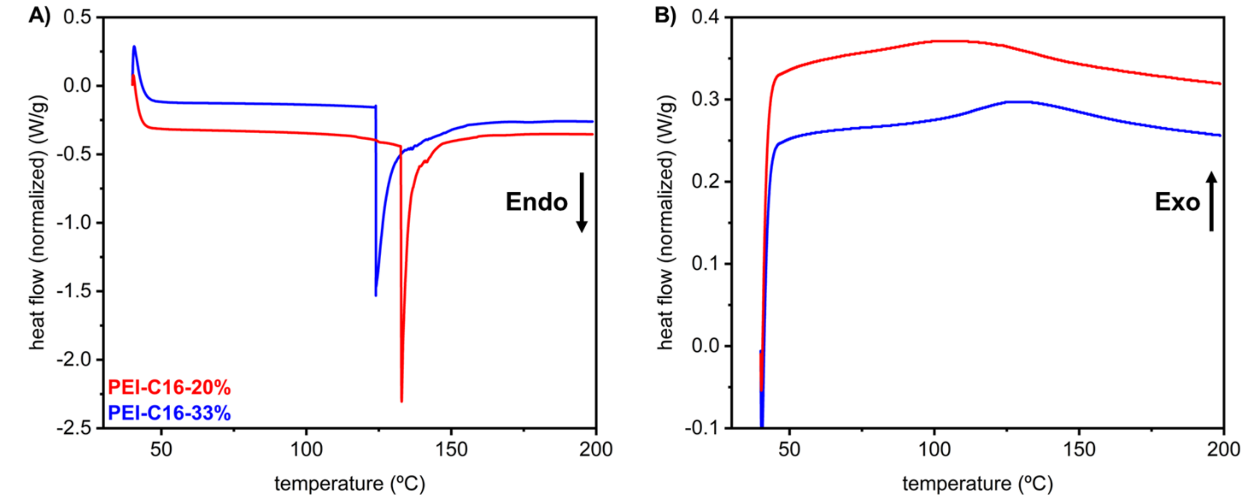

Figure S1. DSC thermograms of **PEI-C16-750K-(33%)** and **(-20%)**: (**A**) heating process (endothermic transition); (**B**) cooling process (exothermic transition).

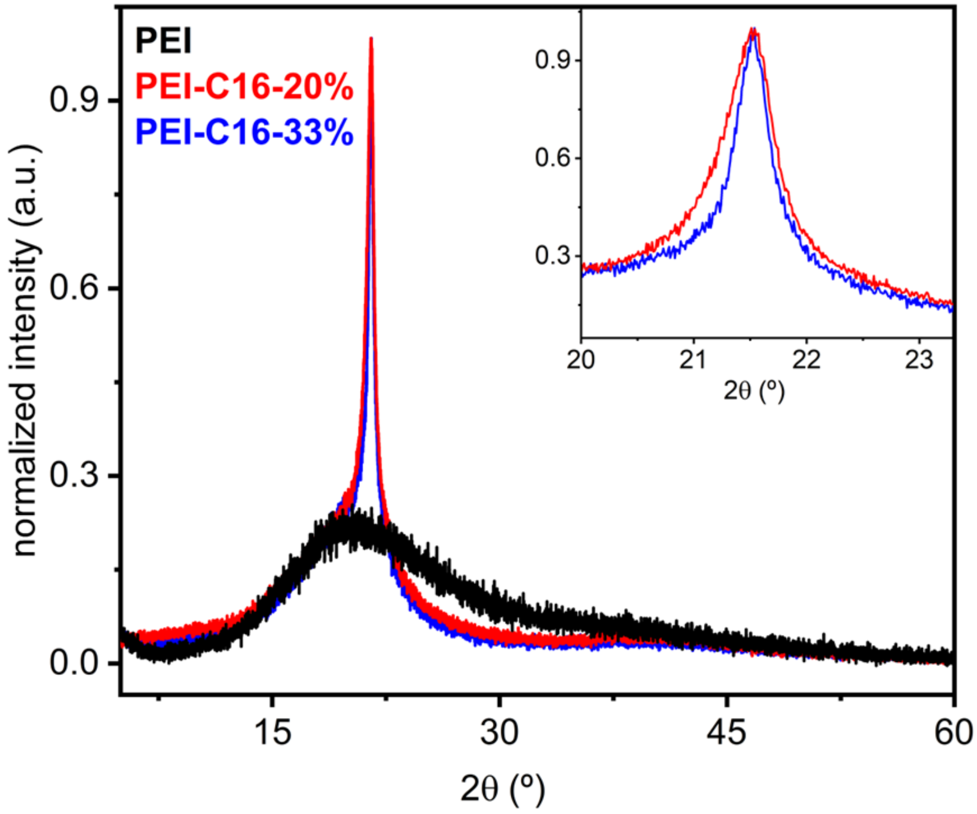

Figure S2. Powder X-ray diffraction patterns (normalized) of **PEI**, **PEI-C16-33%** and **PEI-C16-20%**.

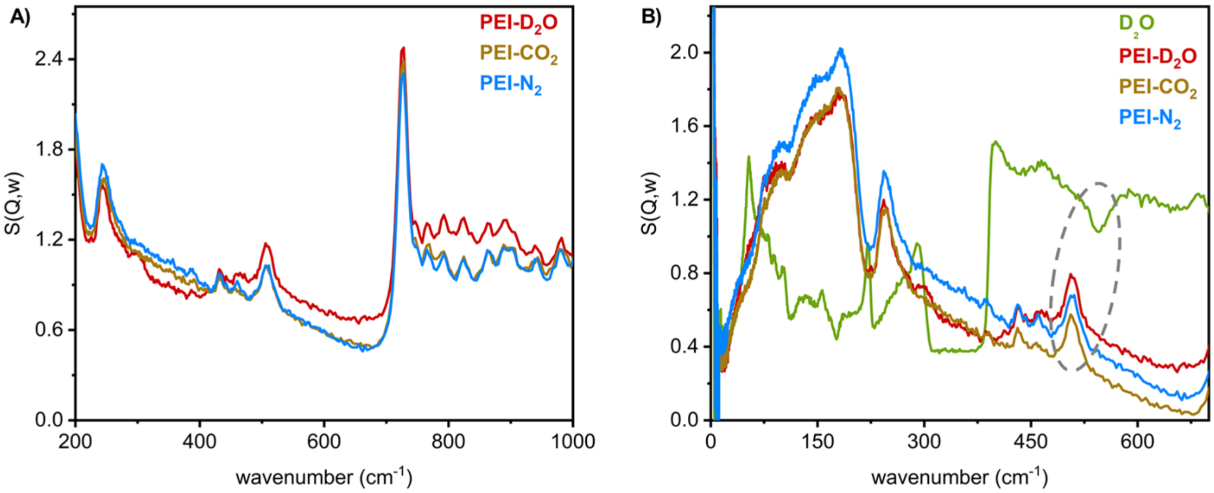

Figure S3. (A) Inelastic neutron scattering analysis of **PEI-C16** untreated (**PEI- N_2**), treated with CO_2 (**PEI- CO_2**) and deuterium oxide exchange reaction (**PEI-D_2O**); (B) Comparative analysis of INS spectra of three samples with D_2O in the frequency range 0-700 cm^{-1}.

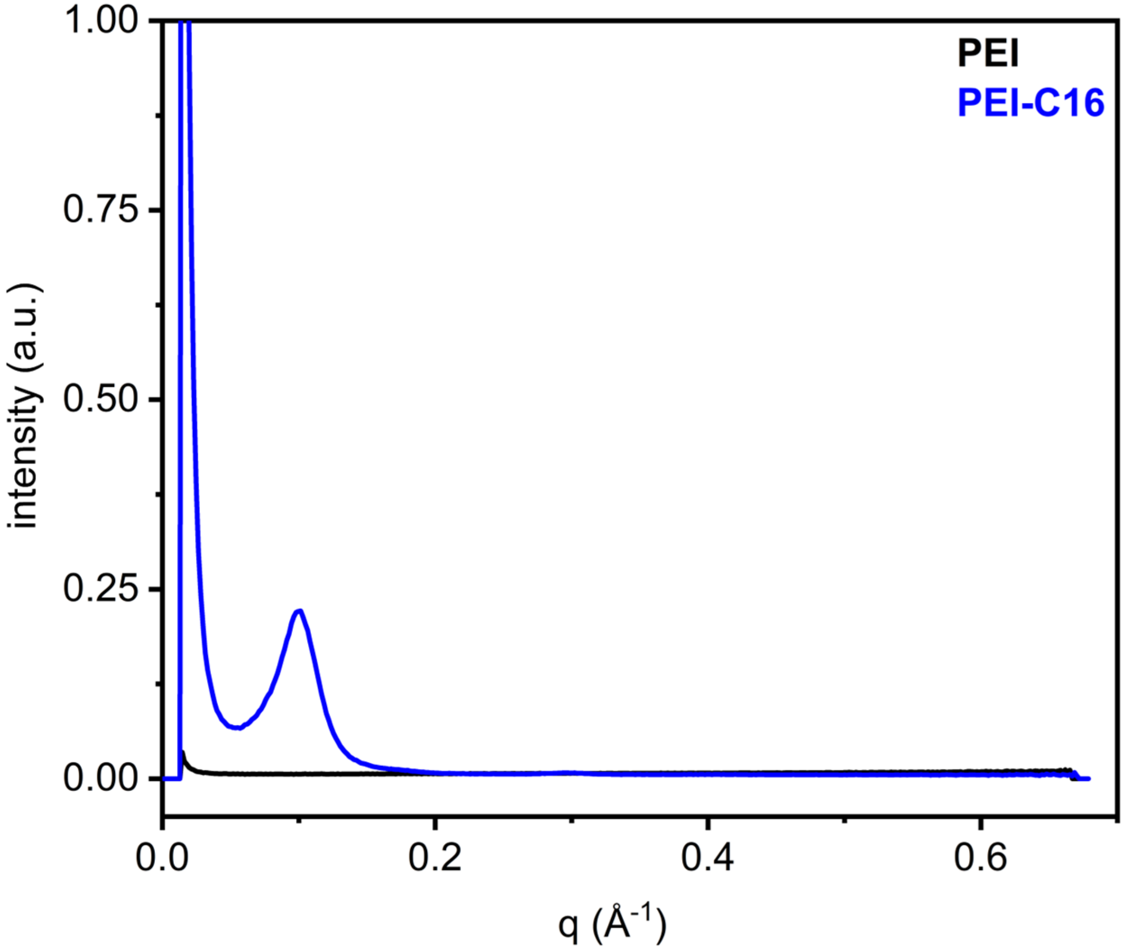

Figure S4. Small angle X-ray Scattering of **PEI-C16** (**PEI-C16-750K-33%**). Distance between sample holder and detector was 350mm.

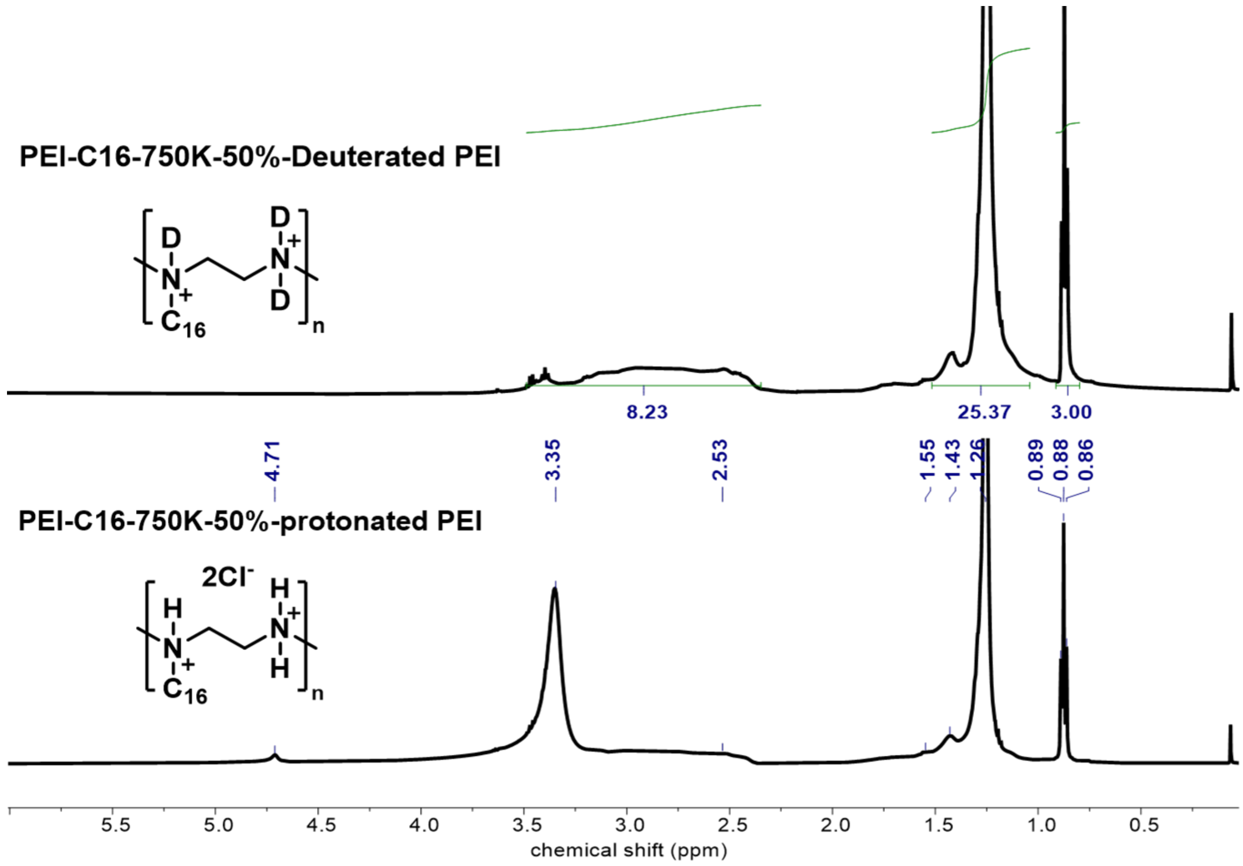

Figure S5. ^{1}H NMR spectra of activated **PEI-C$_{16}$-750K-50%** during base treatment and after adding D_2O (in 0.7 mL $CDCl_3$). The replaceable hydrogens after protonation would effectively be replaced with deuterium resulting in disappearance of sharp resonance peak.

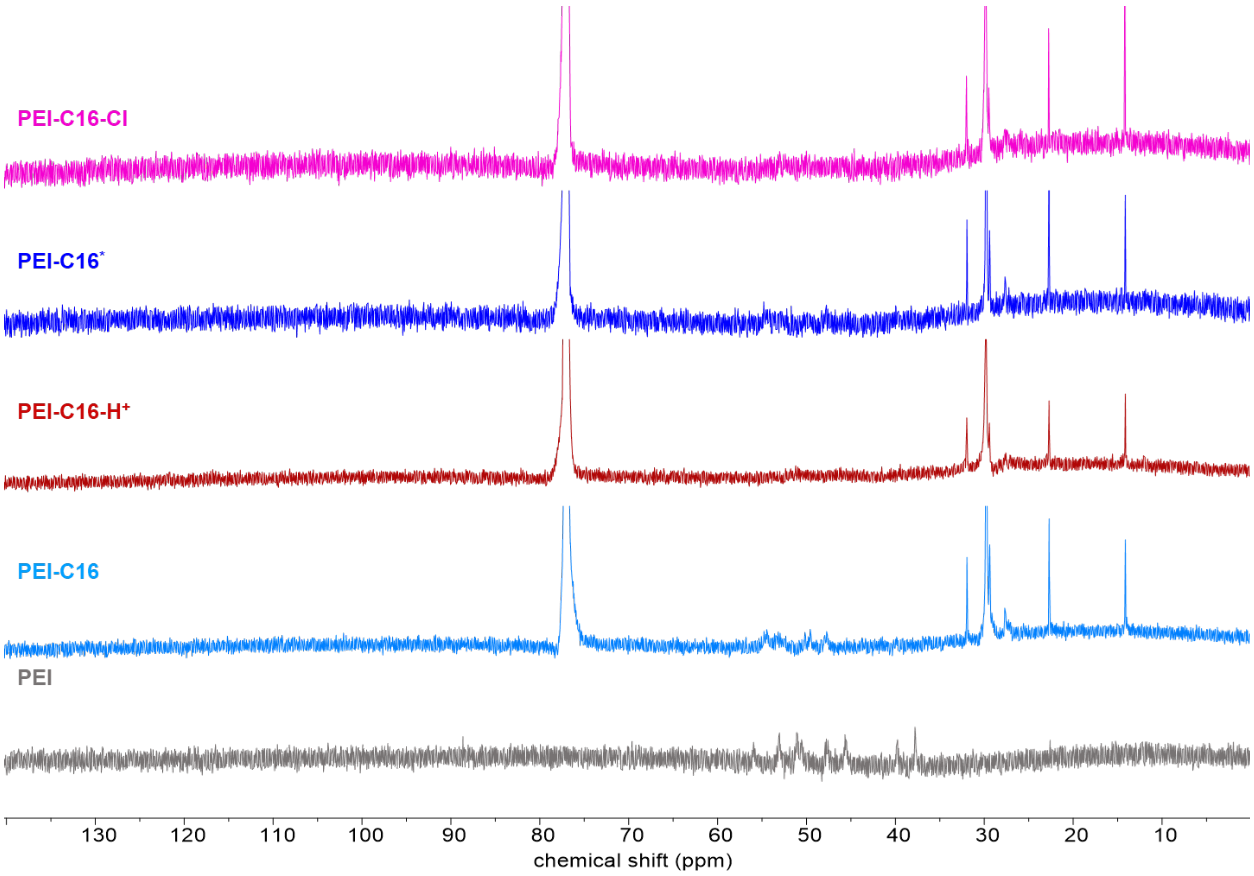

Figure S6. ^{13}C NMR spectra of **PEI-C16** after acidification (in aq. HCl) and desalination of aq. NaCl.

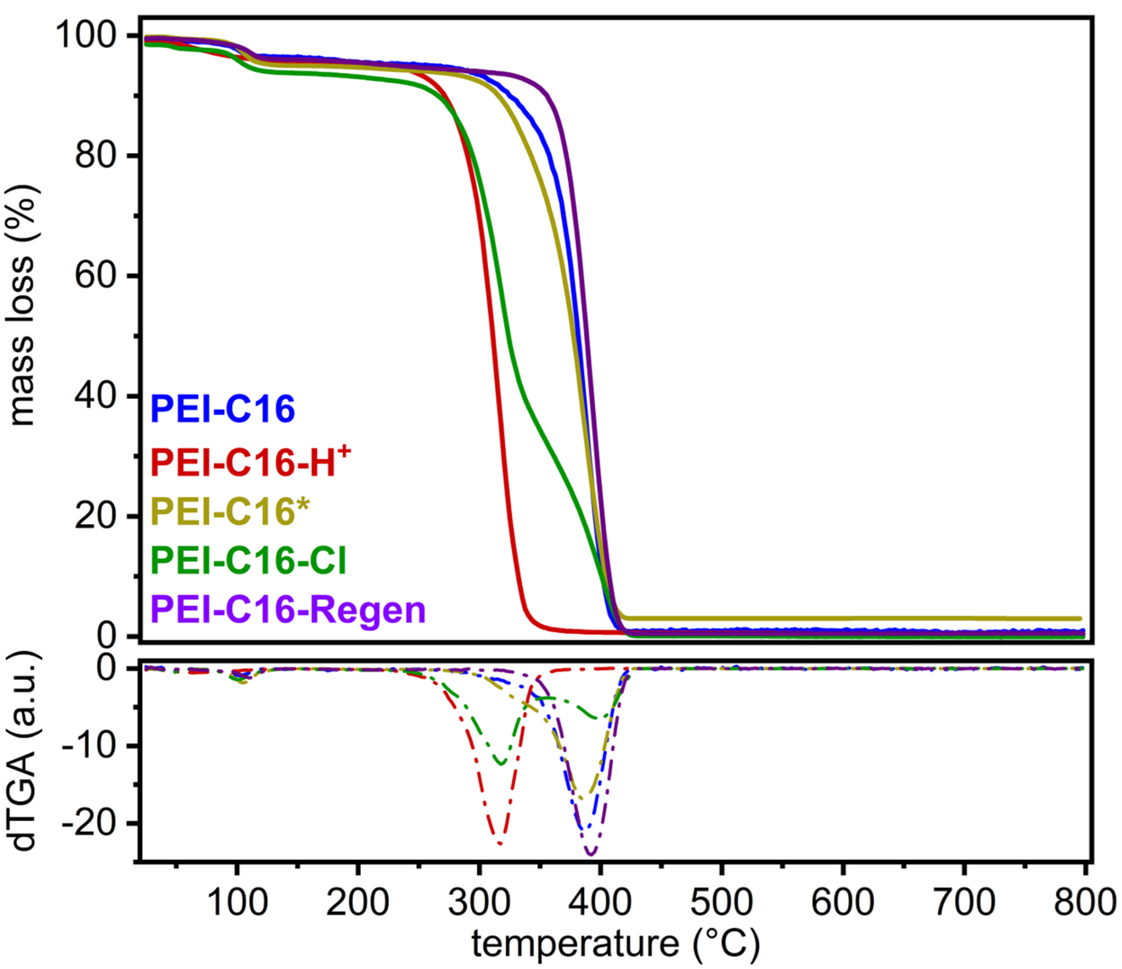

Figure S7. TGA and dTGA (derivative of TGA data points) results for **PEI-C16** collected at a heating rate of 10 K/min. **PEI-C16-H$^+$** treat: **PEI-C16** was treated with aq. HCl (0.1N) solution; **PEI-C16***: **PEI-C16-H$^+$** was treated with aq.NaOH (0.1N) solution; **PEI-C16-Cl**: **PEI-C16*** was used for desalination in aq. NaCl solution; **PEI-C16-Regen**: **PEI-C16-Cl** was treated with aq.NaOH (0.1N) solution.

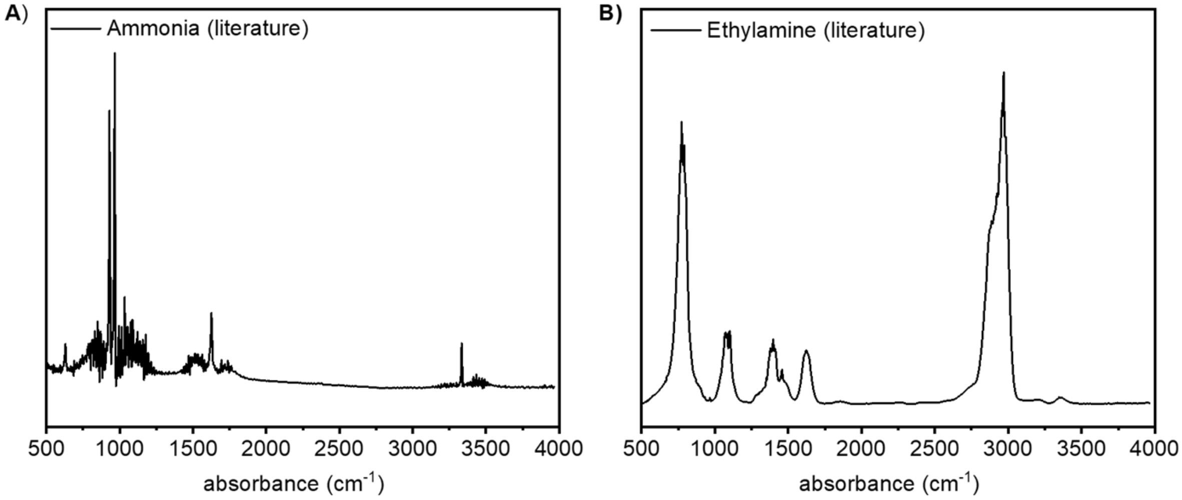

Figure S8. Literature data on IR spectra of evolved ammonia and ethylamine. The IR data points were downloaded and replotted from ref. 2

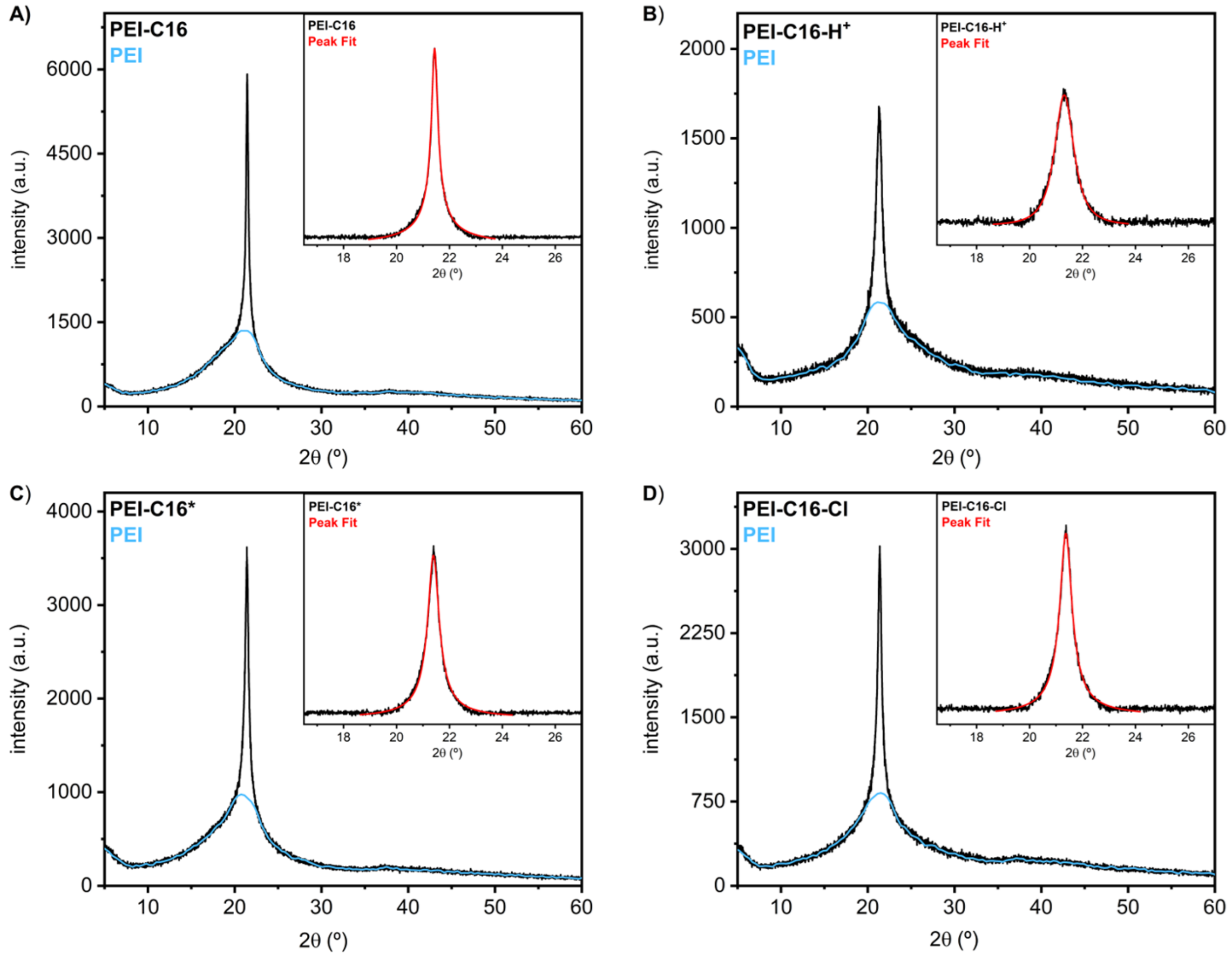

Figure S9. Powder X-ray diffraction patterns of A) **PEI-C16**, B) **PEI-C16-H^{+}**, C) **PEI-C16*** and D) **PEI-C16-Cl**. The insets reveal data after removal of the amorphous contribution (**PEI**) and fit of the peak profile with Pearson-VII function to determine the corresponding FWHM contribution of the C16 chains.

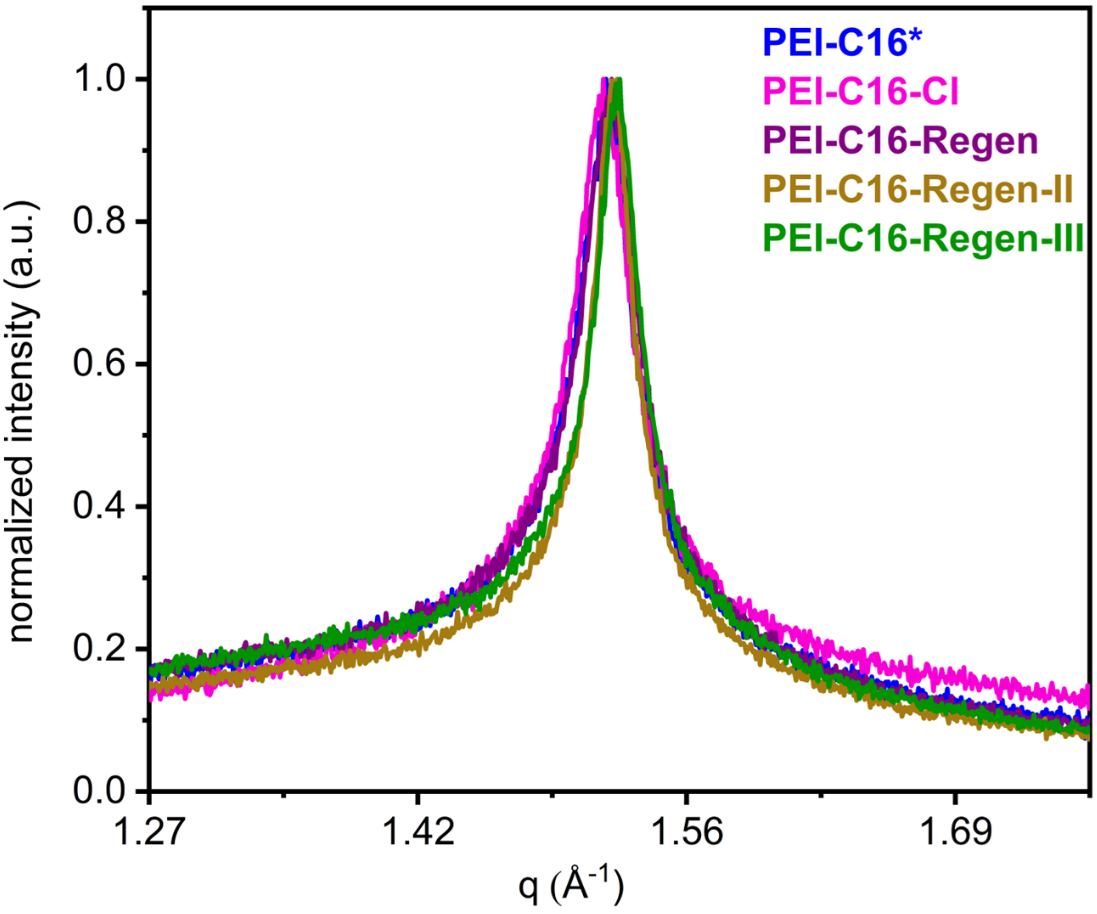

Figure S10. PXRD data of PEI-C16 residues collected from three regeneration cycles along with the chloride reduction data from desalination of aq. NaCl (3 g/L) using the regenerated residues.

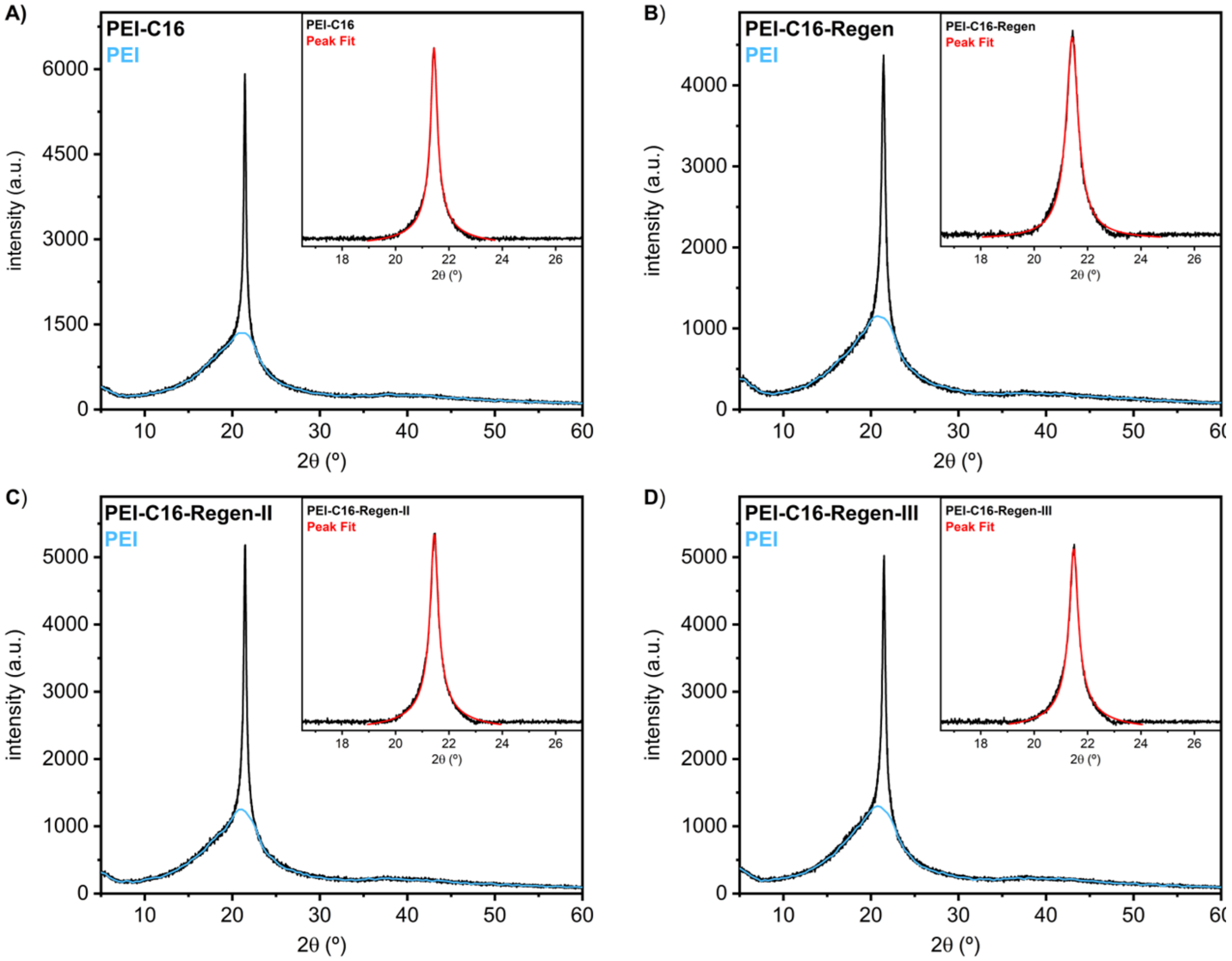

Figure S11. Powder X-ray diffraction patterns of A) **PEI-C16***, B) **PEI-C16-Regen**, C) **PEI-C16-Regen-II** and D) **PEI-C16-Regen-III**. The insets reveal data after removal of the amorphous contribution (**PEI**) and fit of the peak profile with Pearson-VII function to determine the corresponding FWHM contribution of the C16 chains.

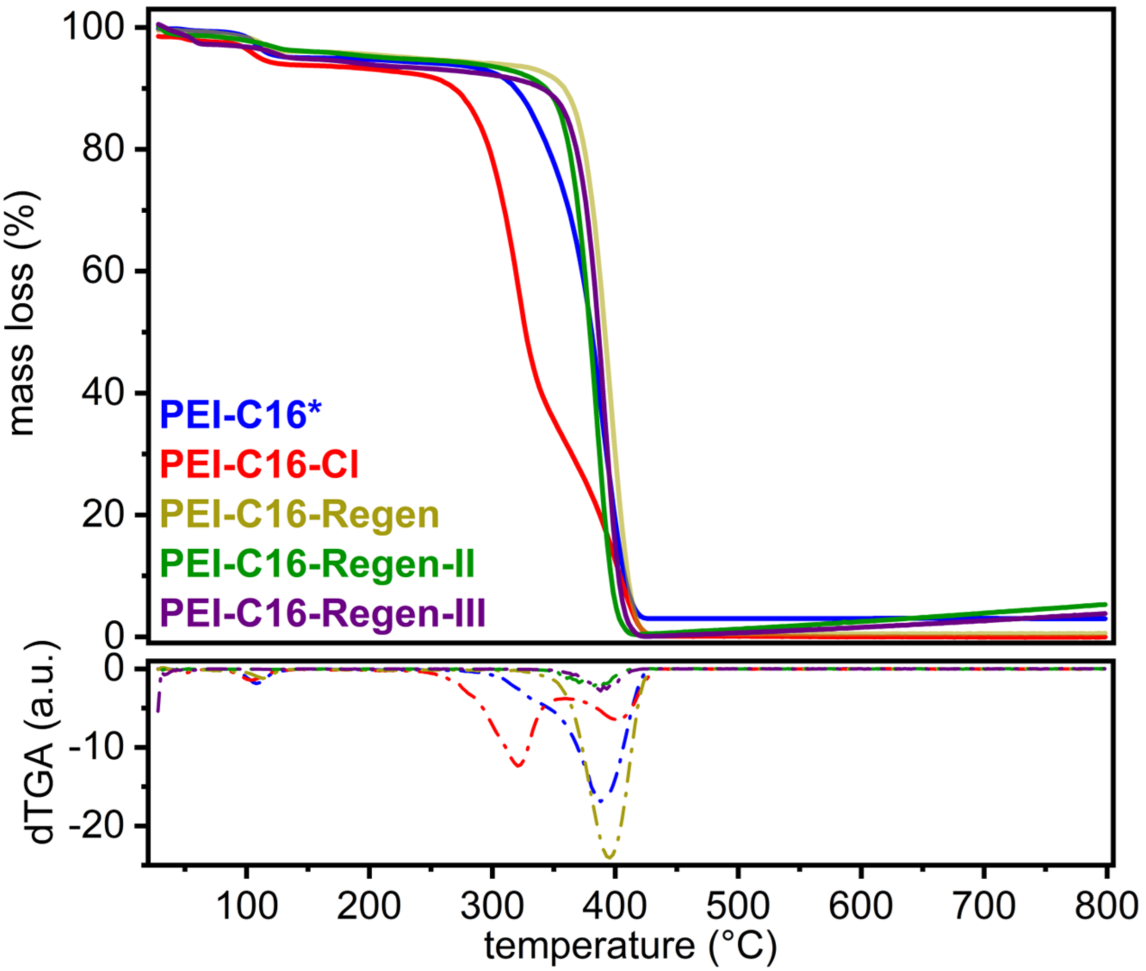

Figure S12. TGA and dTGA (derivative of TGA data points) results for **PEI-C16** collected at a heating rate of 10 K/min. **PEI-C16-Cl**: **PEI-C16** was used for desalination in aq. NaCl solution; **PEI-C16-Regen**: **PEI-C16-Cl** was then treated with aq.NaOH (0.1N) solution; **PEI-C16-Regen-Cl**: **PEI-C16-Regen** was used for desalination in aq. NaCl solution; **PEI-C16-Regen-II**: **PEI-C16-Regen-Cl** was then treated with aq.NaOH (0.1N) solution; **PEI-C16-Regen-II-Cl**: **PEI-C16-Regen-II** was used for desalination in aq. NaCl solution; **PEI-C16-Regen-III**: **PEI-C16-Regen-II-Cl** was then treated with aq.NaOH (0.1N) solution.

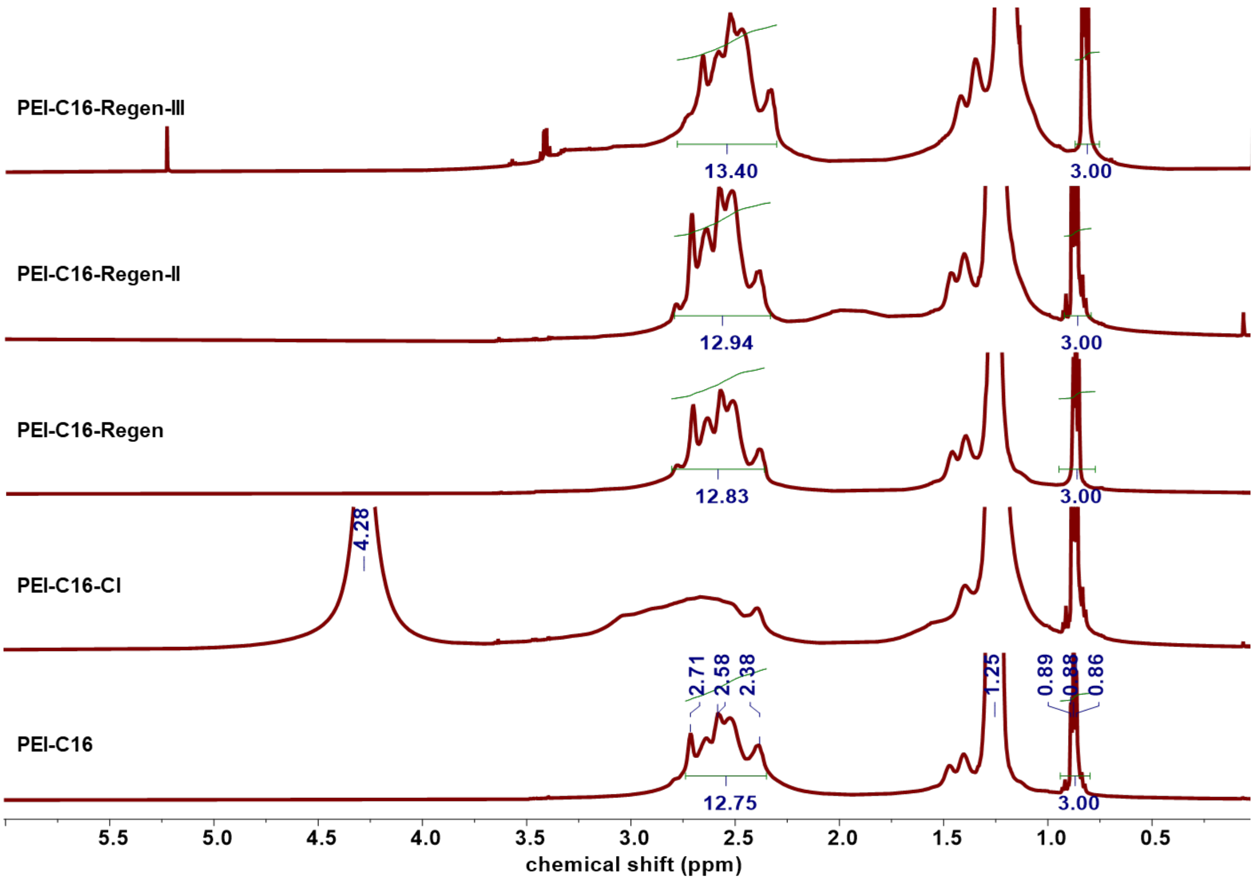

Figure S13. ^{1}H NMR spectra of **PEI-C16, PEI-C16-Cl**, **PEI-C16-Regen**, **PEI-C16-Regen-II** and **PEI-C16-Regen-III**. **PEI-C16-Cl**: **PEI-C16** was used for desalination in aq. NaCl solution; **PEI-C16-Regen**: **PEI-C16-Cl** was then treated with aq.NaOH (0.1N) solution. This procedure of desalination and regeneration was repeated twice.

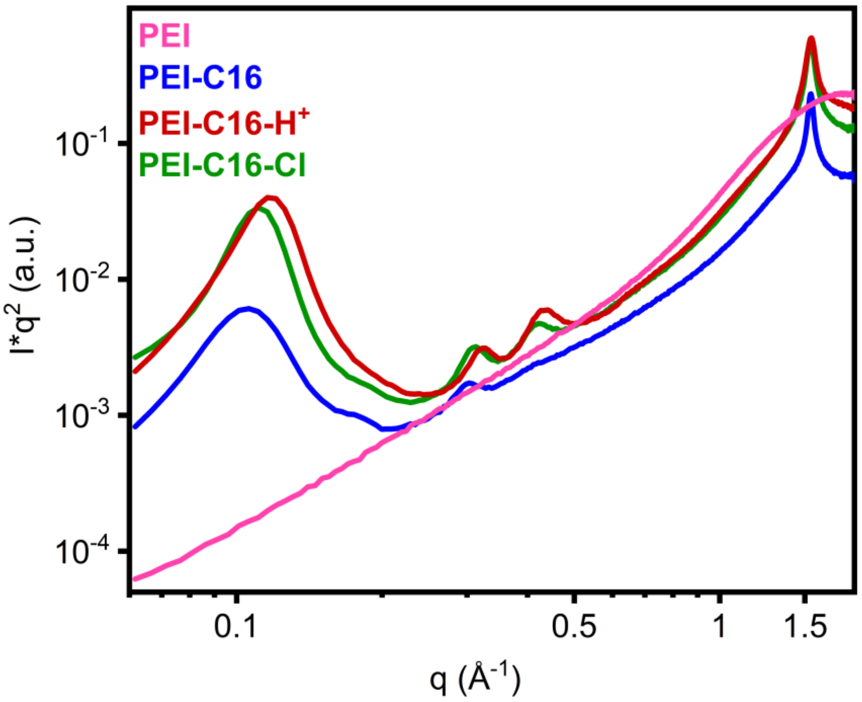

Figure S14. SAXS profiles of PEI, PEI-C16, protonated form (PEI-C16-H+) and after desalination in aq. NaCl solution (PEI-C16-Cl)

REFERENCES

(1) Lima, R. J. S.; Okhrimenko, D. V.; Rudić, S.; Telling, M. T. F.; Sakai, V. G.; Hwang, D.; Barin, G.; Eckert, J.; Lee, J.-W.; Bordallo, H. N. Ammonia Storage in Hydrogen Bond-Rich Microporous Polymers. *ACS Applied Materials & Interfaces* **2020**, *12* (52), 58161-58169. DOI: 10.1021/acsami.0c18855.

(2) Linstrom, P. J.; Mallard, W. G. The NIST Chemistry WebBook: A chemical data resource on the internet. *J. Chem. Eng. Data* **2001**, *46* (5), 1059-1063.
